# First person – Ling Wang

**DOI:** 10.1242/bio.049643

**Published:** 2019-12-05

## Abstract

First Person is a series of interviews with the first authors of a selection of papers published in Biology Open, helping early-career researchers promote themselves alongside their papers. Ling Wang is first author on ‘ NANOG and LIN28 dramatically improve human cell reprogramming by modulating LIN41 and canonical WNT activities’, published in BiO. Ling is a postdoc in the lab of Dr Young Tang at the University of Connecticut, USA, investigating somatic cell reprogramming and mechanisms of induced pluripotency for humans and large farm animal cells.


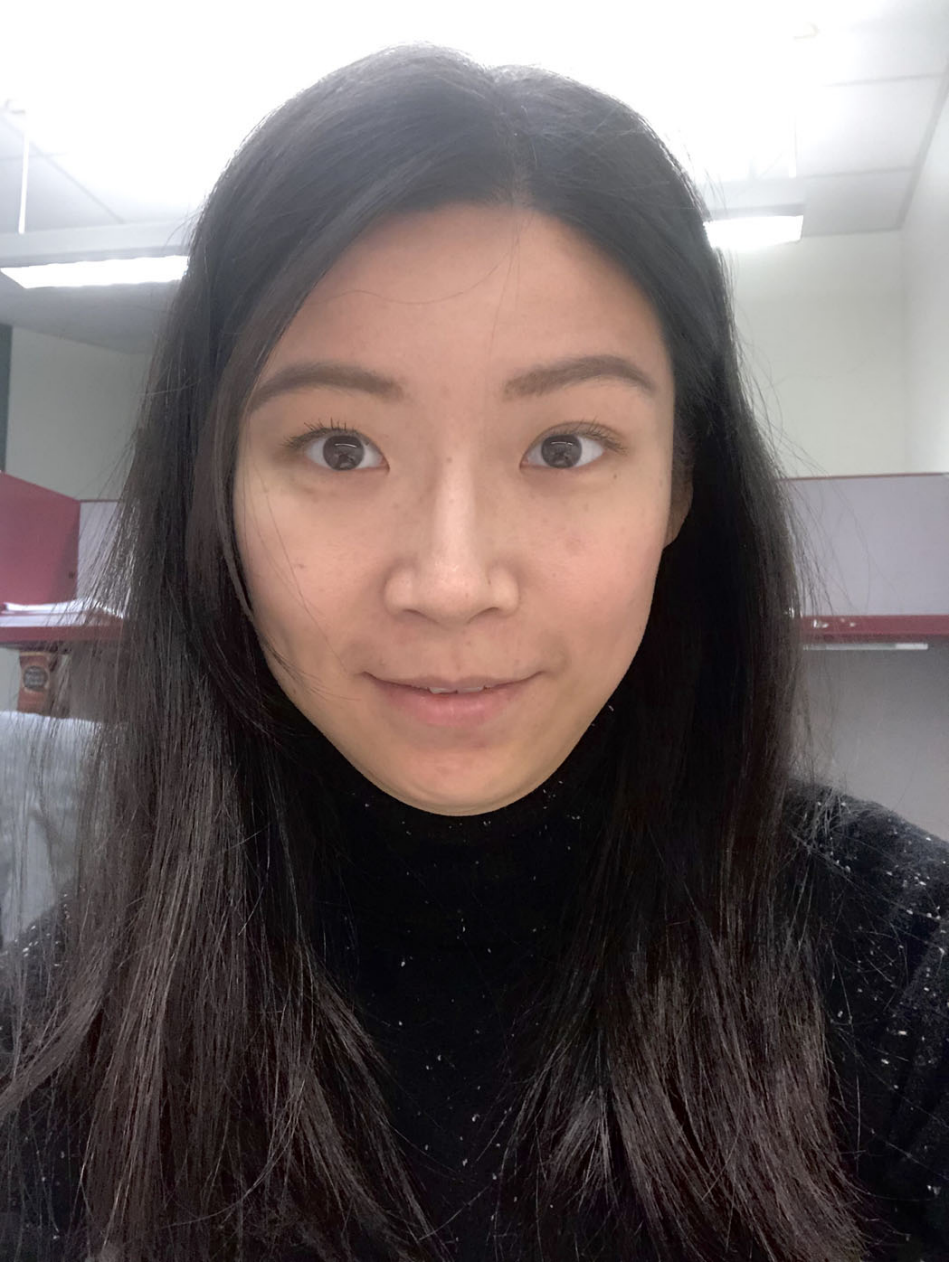


**Ling Wang**

**What is your scientific background and the general focus of your lab?**

Absorbed by the self-renewal and differentiation ability of stem cells, I dived into the research on pluripotent stem cell (PSC) growth regulation and somatic cell reprogramming into induced pluripotent stem cells (iPSCs) in Dr Young Tang's lab at the University of Connecticut. I obtained my PhD degree in animal reproduction this year and am doing a short-term postdoc in the same lab. With my colleagues in the Tang lab, we induce mouse iPSCs to investigate how naïve stage pluripotency (germline chimerism) is established. We also work on improving human iPSC induction in different ways. Our robust human iPSC induction systems [>100-fold increase in efficiency compared with the Yamanaka (conventional OSKM) cocktail] lead us to reveal the key genes and signaling pathways for the enhanced reprogramming. We are now eager to reprogram the somatic cells of large farm animals into iPSCs, which will allow the study of livestock pluripotency regulation and facilitate the breeding of genetically elite agricultural animals.

**How would you explain the main findings of your paper to non-scientific family and friends?**

The stem cells isolated in embryos within a few days after the sperm-egg fertilization can give rise to many mature cell types that make up our body. For an adult, our body cells, such as skin cells, can be induced into these very early embryonic stem cells. The induced cells are called iPSCs. The iPSCs can regenerate any of our tissues or organs with minimum immune suppression because our own cells are used. This technology enables cell-replacement therapies to replace a patient's diseased tissue. But the low induction efficiency of human iPSCs hampers its wide application. We are able to amplify the efficiency by >100-fold with proper combinations of genes and cell culture medium components. This robust system allows for the production of large amounts of patient-specific iPSCs in a short time. We also investigate the genes and signaling pathways core to enhanced reprogramming.

**What are the potential implications of these results for your field of research?**

This system is of importance for the generation of large amounts of iPSCs, especially for patients whose cells might be refractory to reprogramming. In addition to speeding up regenerative medicine therapies, this system may enable the fast generation of multiple isogenic iPSC lines for patients, to account for variations among iPSC lines and genetic background differences among individuals, allowing for more accurate studies on monogenic and polygenic diseases. This reprogramming system may also facilitate precision medicine, which aims for the right dose of the right medications based on genomic information, environment and the lifestyles of the patients.

“In addition to speeding up regenerative medicine therapies, this system may enable the fast generation of multiple isogenic iPSC lines for patients…”

**What has surprised you the most while conducting your research?**

Given their roles in pluripotency regulation, it is reasonable that NANOG and LIN28 synergize in the formation of large numbers of initial colonies with TRA-1-60 expression. But less than 20% of these colonies have homogeneous expression of TRA-1-60, the pluripotent surface maker. The majority (>80%) of these colonies exhibit irregular shapes and have sporadic expression of TRA-1-60. What are the identities of these heterogeneously TRA-1-60-expressing colonies? By inhibiting the canonical WNT signalling at late reprogramming stage, most of these colonies turn into a more mature human embryonic stem cell morphology and have homogeneous and more intense TRA-1-60 expression. The inhibition of canonical WNT also suppressed meso- and endodermal gene activation during reprogramming. These observations suggest that most of NANOG- and LIN28-induced initial colonies are concurrently undergoing WNT-mediated differentiation as they develop!

**Figure:**
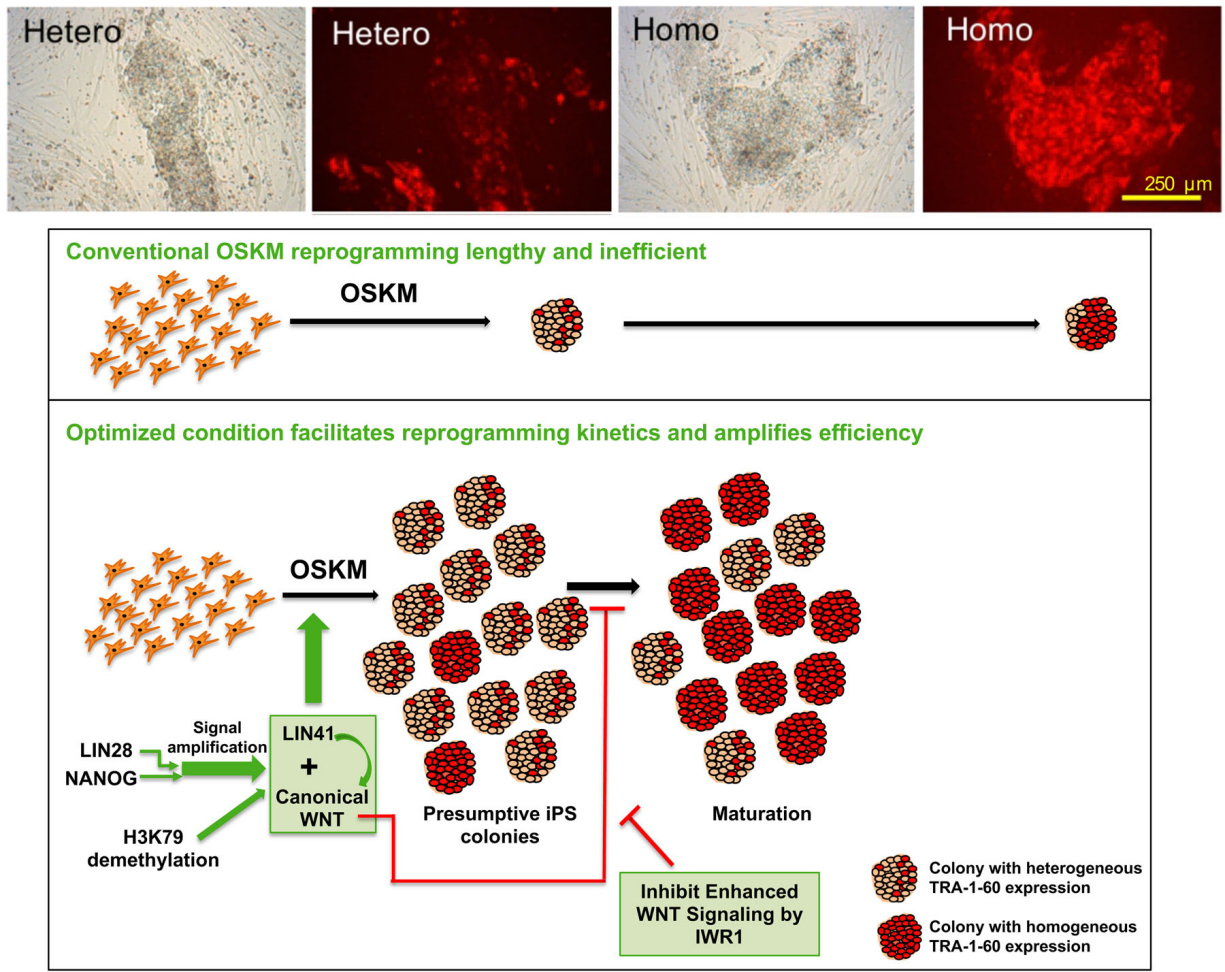
**NANOG and LIN28 synergize in the generation of many colonies with sporadic TRA-1-60 expression (upper left two images). Inhibition of canonical WNT signalling in the late reprogramming stage leads most of the induced colonies to express homogenous and brighter TRA-1-60 (upper right two images).** Graphical abstract of the mechanism of the robust human cell reprogramming optimized in this study (lower panel).

We further found that NANOG and LIN28-stimulated canonical WNT signaling has a biphasic role, since iPSC colony number greatly reduces if the WNT is inhibited from the beginning of reprogramming.

**What, in your opinion, are some of the greatest achievements in your field and how has this influenced your research?**

Somatic cell nuclear transfer (SCNT) by Sir John Gurdon in frogs, the first cloned animals, demonstrates that somatic cells carry the nuclei with complete genetic materials to develop into a normal animal. The success in Dolly the sheep confirms this principle in mammals. Dr Shinya Yamanaka's research into human iPSCs reveals the four genes sufficient to turn the somatic cells back to embryonic pluripotent state.

On the basis of these ground-breaking achievements, and other groups' reported factors to promote reprogramming, we examined different combinations of genes and small chemicals and found two combinations (Yamanaka cocktail+NANOG+LIN28+iDOT1L+IWR1 and Yamanaka cocktail+NANOG+ +LIN41+iDOT1L+IWR1) that could induce hundreds of iPSC colonies in a short time.

**What changes do you think could improve the professional lives of early-career scientists?**

Active communication with peer researchers and professors. By attending academic conferences, we can present our findings as well as learn about the latest advancement in the research field. This will help us stay focused on the big picture and ultimate goals, not getting buried in the details. It will also guide us in asking the most interesting questions.

**What's next for you?**

I am excited to employ our optimized iPSC derivation system for more mechanistic studies on robust human cell reprogramming. It is also exciting to bring this system to translational research. I look forward to all the possible employment opportunities (hopefully) in related research fields.

**Who impacted you most in your doctoral training?**

I benefitted a lot from my doctoral advisory committee: my major advisor, Dr Young Tang, and Dr Xiuchun (Cindy) Tian, Dr Kristen Govoni, Dr Theodore Rasmussen and Dr Sarah Reed. They have always been encouraging and patiently provide advice on my project designs. Furthermore, they guide me towards insights from my research findings and help to push my projects further.
